# Laboratory evaluation of the broad-spectrum antibacterial efficacy of a low-irradiance visible 405-nm light system for surface-simulated decontamination

**DOI:** 10.1007/s12553-023-00761-3

**Published:** 2023-06-14

**Authors:** Lucy G Sinclair, Laura R Dougall, Zornitsa Ilieva, Karen McKenzie, John G Anderson, Scott J MacGregor, Michelle Maclean

**Affiliations:** 1 The Robertson Trust Laboratory for Electronic Sterilisation Technologies, Department of Electronic & Electrical Engineering, University of Strathclyde, Glasgow, UK; 2 Department of Biomedical Engineering, University of Strathclyde, Glasgow, UK

**Keywords:** 405nm light, Violet-blue light, Antibacterial, Environmental decontamination system, Environmental decontamination, Bacteria

## Abstract

**Purpose:**

Lighting systems which use visible light blended with antimicrobial 405-nm violet-blue light have recently been developed for safe continuous decontamination of occupied healthcare environments. This paper characterises the optical output and antibacterial efficacy of a low irradiance 405-nm light system designed for environmental decontamination applications, under controlled laboratory conditions.

**Methods:**

In the current study, the irradiance output of a ceiling-mounted 405-nm light source was profiled within a 3×3×2 m (18 m^3^) test area; with values ranging from 0.001-2.016 mWcm^-2^. To evaluate antibacterial efficacy of the light source for environmental surface decontamination, irradiance levels within this range (0.021-1 mWcm^-2^) at various angular ($\Delta$ ϴ=0-51.3) and linear (∆s=1.6-2.56 m) displacements from the source were used to generate inactivation kinetics, using the model organism, *Staphylococcus aureus*. Additionally, twelve bacterial species were surface-seeded and light-exposed at a fixed displacement below the source (1.5 m; 0.5 mWcm^-2^) to demonstrate broad-spectrum efficacy at heights typical of high touch surfaces within occupied settings.

**Results:**

Results demonstrate that significant (P≤0.05) inactivation was successfully achieved at all irradiance values investigated, with spatial positioning from the source affecting inactivation, with greater times required for inactivation as irradiance decreased. Complete/near-complete (≥93.28%) inactivation of all bacteria was achieved following exposure to 0.5 mWcm^-2^ within exposure times realistic of those utilised practically for whole-room decontamination (2-16 h).

**Conclusion:**

This study provides fundamental evidence of the efficacy, and energy efficiency, of low irradiance 405-nm light for bacterial inactivation within a controlled laboratory setting, further justifying its benefits for practical infection control applications.

## Introduction

The use of violet-blue visible light technology for the inactivation of bacteria has emerged as an area of increasing research interest. Although less lethal than ultraviolet light, violet-blue visible light, with particular emphasis on 400-420 nm wavelengths, has proved effective for the inactivation of a range of bacterial species, and can provide an alternative or supplementary method of decontamination, in an area where novel technologies are increasingly required due to the challenges of antibiotic and disinfectant resistance.

Investigations into the mechanism of action indicate that photodynamic inactivation by violet-blue light exposure occurs as a result of the photoexcitation of intracellular porphyrin molecules within the exposed bacteria resulting in production of reactive oxygen species, such as singlet oxygen, which can damage and inactivate bacterial cells [1–4]. Previous studies have shown wavelengths in the region of 400-420 nm to be most effective for bacterial inactivation, with optimal activity at 405 nm [5, 6]. This peak in activity correlates with the absorption maximum of porphyrin molecules, termed the Soret band, being in this wavelength region [7]. Light of these wavelengths have a wide spectrum of activity and its ability to inactivate a range of micro-organisms, including bacteria, fungi and viruses, presented as aerosols, in liquid suspension and on surfaces, has been widely demonstrated [5, 6, 8–17]. In terms of its bactericidal capabilities, successful inactivation by 405-nm light has been demonstrated for a wide range of Gram-positive and Gram-negative pathogenic bacterial species including those associated with healthcare and clinical infections, such as *Staphylococcus aureus*, *Clostridium difficile*, *Acinetobacter baumannii*, *Pseudomonas aeruginosa, Helicobacter pylori, Chlamydia* and *Mycobacterium* species [2, 8–10, 18–20]; bacteria associated with foodborne infection including *Listeria monocytogenes*, *Escherichia coli*, *Campylobacter jejuni* and *Salmonella* species [5, 8, 11]; and oral periodontal pathogens [21, 22].

In efforts to address the challenges associated with environmental decontamination, a novel disinfection technology has been developed which comprises a ceiling-mounted blended light system that produces enhanced levels of low irradiance (<1 mW cm^-2^) antimicrobial 405-nm light. Termed the 405-nm light environmental decontamination system (EDS), these systems emit intensities of light which are inherently bactericidal at levels which are non-detrimental to mammalian cells, meaning the technology can be used to provide a safe and continuous decontamination effect to the air and exposure surfaces of occupied whole-room environments [23]. This is uniquely advantageous over other whole-room decontamination technologies such as ultraviolet light which, although has a much higher germicidal efficiency than that of 405-nm light, can induce long-term material degradation and damage to human skin and eye tissues [24, 25] and thus is generally limited to episodic and short-term use in unoccupied environments.

First described as a potential method of environmental decontamination by Maclean et al. [8], the system has since been commercially licensed for this purpose, with clinical evidence demonstrating its ability to successfully reduce environmental bacterial contamination levels within healthcare settings including isolation rooms and outpatient clinics by up to 86% when used in conjunction with standard hygiene protocols [23, 26–28] and, more recently, subsequently reduce rates of surgical site infections when used in orthopaedic operating theatres [29]. In the latter, the authors found that this bactericidal effect occurred not only in the room of use, but also in an adjacent room with a shared air circulatory system, highlighting the efficacy of 405-nm light EDS units for *in situ* inactivation of airborne bacteria [29].

For use in occupied indoor environments it is essential that the violet-blue light wavelengths employed are delivered at a sufficiently low irradiance that they are within levels considered safe for continuous human exposure in accordance with international guidelines [30]. Previous laboratory-based studies on the bactericidal properties of violet-blue light have typically used relatively high irradiance light sources (typically 3-200 mW cm^-2^) in order to demonstrate a rapid inactivation effect [2, 8–10, 18]. Although the clinical studies, previously mentioned, have established that a reduction in general bacterial contamination levels can be achieved utilising low irradiance light, there is as yet no definitive information on the bactericidal efficacy of low irradiance 405-nm light on individual bacterial pathogens. In these previous clinical studies, conducted in dynamic hospital environments, although a general disinfection effect was observed on the exposed surfaces around the room, quantitative data demonstrating inactivation kinetics of individual environmental pathogens could not be obtained [23, 26–29]. In addition, the range of irradiance levels which are likely to illuminate the air and exposed surfaces within a typical room setting is yet to be fully assessed, and so the efficacy of 405-nm light for the inactivation of environmental pathogens at these irradiance levels is widely unknown.

Accordingly, the focus of the present study was to characterise the optical irradiance output profile of low irradiance 405-nm light systems within a typical room setting, and quantify, for the first time, the bactericidal effect of 405-nm light at these irradiances for the inactivation of low-density populations of surface-seeded bacteria, comparable with typical contamination levels found on environmental surfaces. A range of significant bacterial pathogens associated with hospital-acquired and foodborne infections were investigated in order to generate inactivation curves of known bacteria, thus building a better understanding of the exposure times required for inactivation of these bacterial pathogens when found contaminating the environment. Differences in inactivation kinetics, and how bactericidal efficacy is affected spatially by varying irradiance exposure levels, was investigated, with particular focus on the bacterium *Staphylococcus aureus*, selected due to its significance as a causative agent of both healthcare-associated and foodborne infection. These findings will provide a comprehensive laboratory evaluation of 405-nm light systems for environmental decontamination and present evidence of its broad-spectrum antibacterial efficacy at the low irradiance levels which must be employed by 405-nm light EDS units in order for it to comply with exposure safety guidelines.

## Materials and methods

### Light source

The light source used for irradiance profiling and exposure of bacteria was a ceiling-mounted 405-nm light EDS prototype unit which consisted of a matrix of 405-nm light-emitting diode (LED) arrays (for antimicrobial activity), and white LEDs (for light blending), covered by an optical lens system comprising a Fresnel lens and diffuser, which are positioned to aid in light distribution and scatter, respectively [31, 32]. For operation, the arrays were driven by a low-voltage power supply 270 W (15 V at 18 A), and were bonded to an aluminium heatsink in order to minimize the build-up of heat. For all experimental testing, the system was operated in ‘blue-only’ mode (with the white LEDs switched off), and irradiance was measured in mW cm^-2^, using a radiant optical power meter (Model 70260; L.O.T. Oriel Instruments Ltd, USA) and a photodiode detector (Model IZ02413, L.O.T. Oriel Instruments Ltd, USA).

The applied dose was calculated using the equation:

$$Dose \left(J {cm}^{-2}\right)=Irradiance \left(W {cm}^{-2}\right)\times Exposure\;time\;(seconds)$$

## Optical characterisation of the light source

**Fig. 1:**
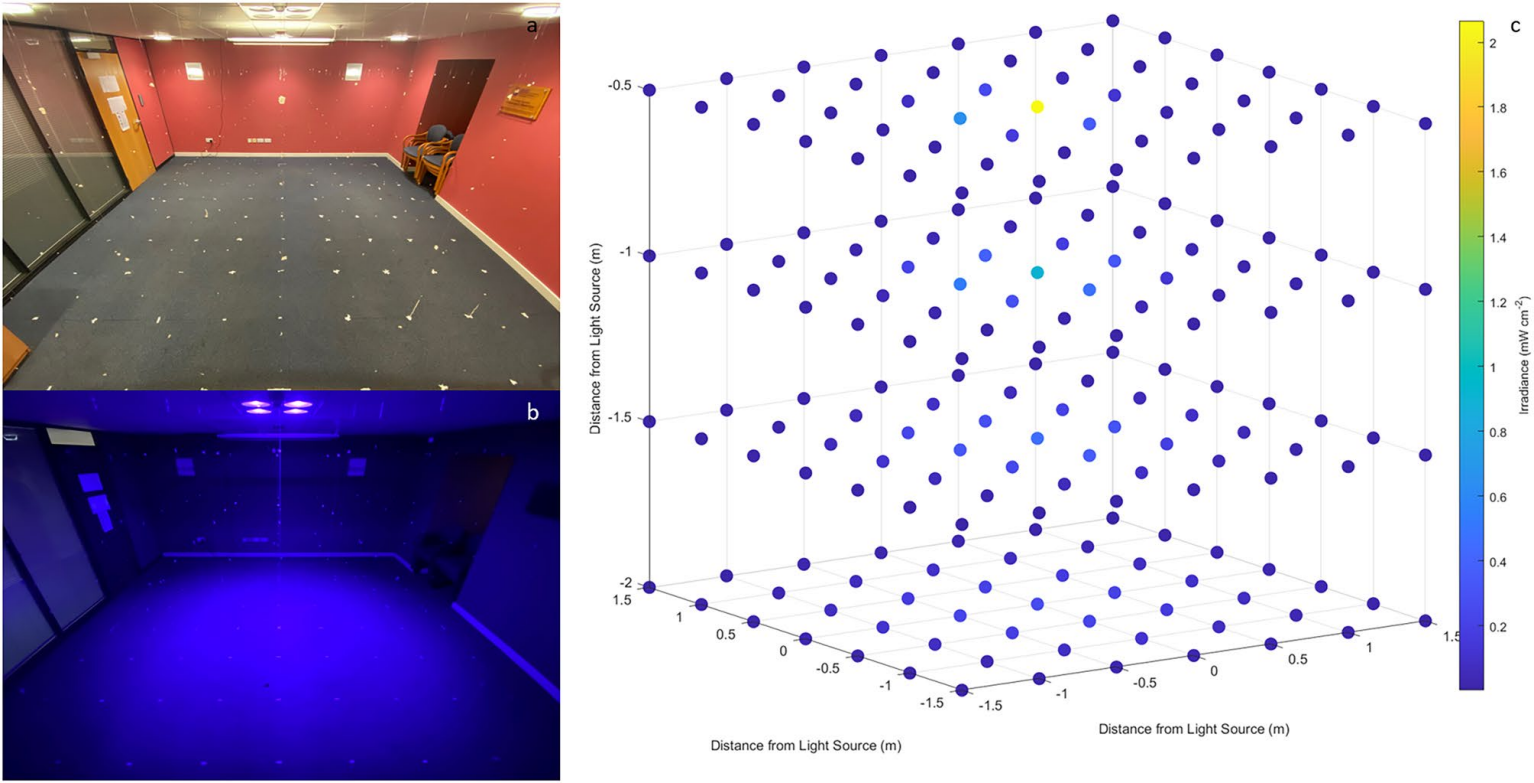
3D irradiance output profiling of a ceiling-mounted low irradiance 405-nm light source designed for whole-room decontamination purposes: **a** the light source installed in the ceiling centre of a 3 × 3 × 2 m sized room; **b** the system operating in ‘blue-only’ mode with standard room lighting switched off for irradiance measurement; and **c** irradiance measurements modelled on MATLAB R2022a

A 3D optical irradiance output profile of the 405-nm light EDS was produced within a 3 × 3 × 2 m area in a vacant room in the University of Strathclyde, Glasgow, UK. The light source was installed in place of a ceiling tile in the centre of this area (Fig. 1a).

For commercial use as an overhead light source, the system incorporates white LEDs in conjunction with the antimicrobial blue LEDs to ensure that the illumination output is predominantly white and blends with standard room lighting such that there is no visual disturbance to room occupants. However, for the purposes of this study, the light source was used in ‘blue-only’ mode (Fig. 1b) to ensure that the inactivation data gathered was accounted for solely by inactivation due to the antimicrobial blue lighting component only. Irradiance measurements were taken at approximately 0.5 m intervals in X, Y and Z directions, presented using MATLAB R2022a in Fig. 1c, which were then analysed using OriginPro v2022.

For further analysis of irradiance distribution, measured irradiance values were plotted as a function of both linear and angular displacement from the light source. The linear displacement (∆s) of each measured point from the light source was calculated by applying Pythagoras theorem, as follows:

$$\Delta s=\sqrt{{\left(distance\in X\right)}^{2}+{\left(distance\in Y\right)}^{2}+{\left(distance\in Z\right)}^{2}}$$

The angular displacement ($\Delta$ ϴ) of each measured point in X and Y directions from the light source was then calculated using the tangent trigonometric function, as follows:

$$\Delta \varnothing ={tan}^{-1}\left(\frac{distance\in X/Y}{distance\in Z}\right)$$

### Culture and preparation of bacteria

The microorganisms used in this study were a range of twelve hospital-acquired and foodborne bacterial pathogens. *Staphylococcus aureus* NCTC 4135, *Staphylococcus epidermidis* NCTC 11964, *Listeria monocytogenes* NCTC 11994, *Escherichia coli* NCTC 9001, *Acinetobacter baumannii* NCTC 12156, *Klebsiella pneumoniae* NCTC 9633, *Salmonella enterica* NCTC 4444 and *Shigella sonnei* NCTC 12984 were obtained from the National Collection of Type Cultures (NCTC), Colindale, London, UK. *Pseudomonas aeruginosa* LMG 9009, *Enterococcus faecium* LMG 11423, *Enterobacter cloacae* LMG 2783 and *Yersinia enterocolitica* LMG 7899 were obtained from The Belgian Co-ordinated Collections of Microorganisms/Laboratorium voor Microbiologie, Universiteit Gent (BCCM/LMG). Bacterial isolates were stored at 4°C on agar slopes until experimental use. For culture, bacteria were inoculated into 100 mL nutrient broth (Oxoid UK), with the exception of *L. monocytogenes* and *E. faecium* which was cultured in tryptone soya broth (Oxoid, UK). Broths were incubated at 37°C for 18 h under rotary conditions (120 rpm), and then centrifuged at 3939×g for 10 min, with the resultant pellet re-suspended in 100 mL phosphate buffered saline (PBS; Oxoid, UK). Bacterial suspensions were then serially diluted in PBS to 10^3^ CFU mL^-1^ for experimental use, and 100 µL volumes were aseptically spread onto the surface of 90 mm diameter nutrient agar plates to provide a starting population of approximately 100-300 colony forming units (CFU) per plate (1.6-4.7 CFU cm^-2^) for each microorganism.

### Light exposure of microbial cultures

#### Inactivation kinetics of bacteria exposed to 405-nm light EDS

To demonstrate the broad-spectrum bactericidal efficacy of the low irradiance 405-nm light system, twelve bacterial species were exposed to increasing doses of 405-nm light on a surface directly below the light source at a distance of approximately 1.5 m; selected as representative of the typical heights of high touch surfaces within public areas. The irradiance produced from the 405-nm light EDS at this distance was found to be 0.5 ± 0.02 mW cm^-2^. Seeded plates (with the lids removed) were exposed to increasing durations of 405-nm light for up to 16 h.

#### Effect of exposure distance and irradiance on inactivation efficacy

To determine how the irradiance output of the 405-nm light EDS affects bactericidal efficacy, inactivation kinetics of *S. aureus*, were established using irradiance levels ranging from 0.05-1 mW cm^-2^. Seeded plates (with the lids removed) were positioned directly under the light source and were exposed to increasing doses of 405-nm light at approximate irradiances of 0.05, 0.15, 0.25, 0.5 and 1 mW cm^-2^.

To determine how distance from the 405-nm light EDS affects bactericidal efficacy, inactivation kinetics of *S. aureus* selected as a model organism, were established for exposures to the 405-nm light EDS across a radius of 2 m measured from the centre of the light source. Seeded plates (with the lids removed) were positioned approximately 1.6 m below the light source, and were situated in 0.1 m intervals from directly under the light source (∆s = 1.6 m; $\Delta$ ϴ = 0) up to a distance of 2 m radially (∆s = 2.56 m; $\Delta$ ϴ = 51.3), with irradiance measured at each fixed position. For each independent experiment, samples were exposed for durations of 4, 8 and 24 h.

#### Enumeration and statistical analysis

All experiments were performed in triplicate for each exposure time (n = 3), with control plates exposed to standard laboratory lighting. Post-exposure, the lids were replaced and the seeded agar plates were incubated at 37°C for 18 h. The viable bacterial colony-forming unit counts per plate (CFU plate^-1^) were then enumerated. Results represent the mean values of triplicate replicates, and are reported as the percentage of surviving or reduced CFU plate^-1^, as compared to the equivalent non-exposed control samples. Significant differences in experiments were calculated using one-way ANOVA at the 95% confidence interval and P≤0.05, using MINITAB Release 16.

## Results

### Optical characterisation of the light source

**Fig. 2:**
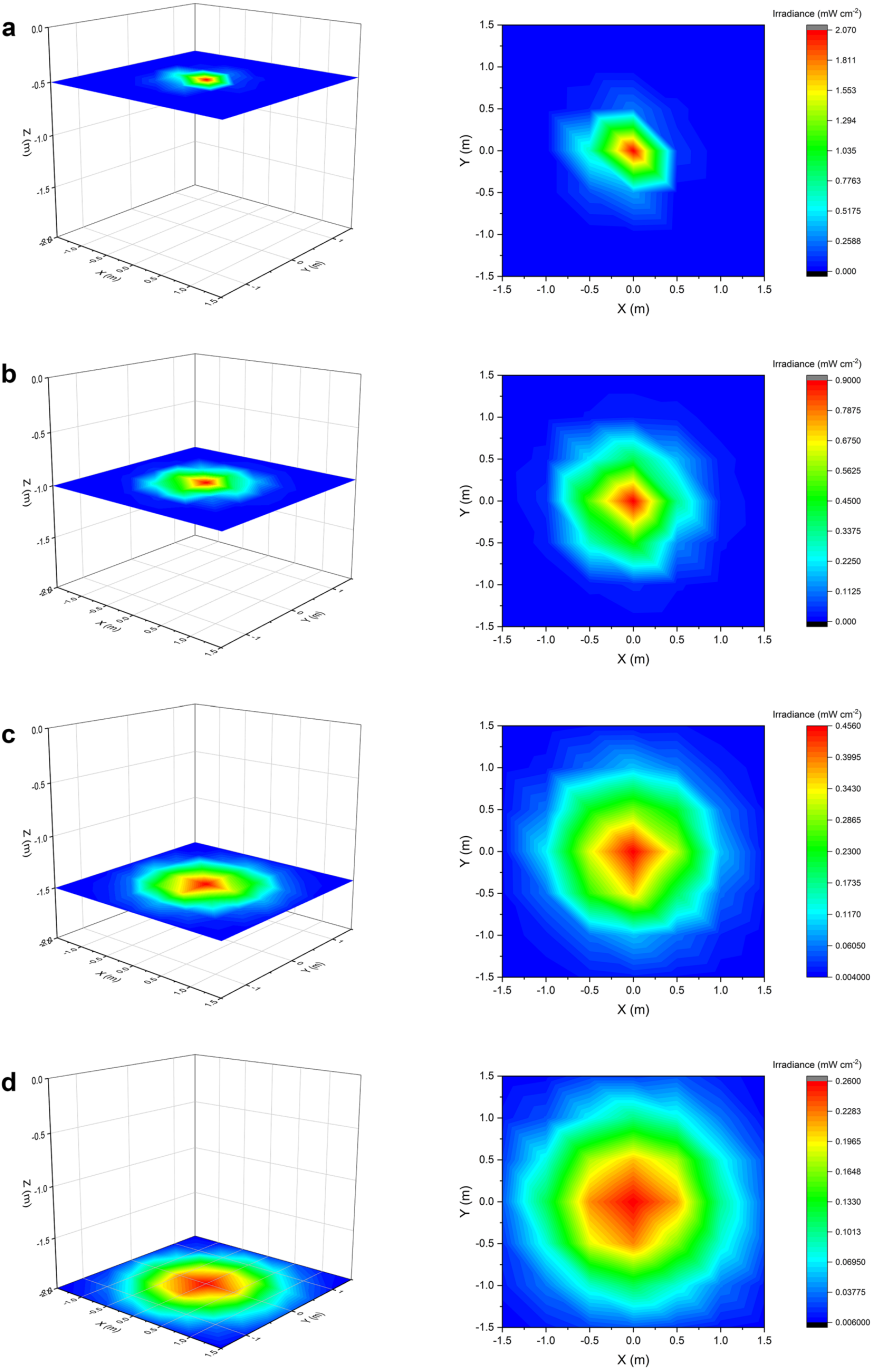
Irradiance distribution produced by ceiling-mounted low-irradiance 405-nm light EDS at distances of **a** 0.5 m, **b** 1 m, **c** 1.5 m and **d** 2 m in the Z direction (please note that the scale is different for each graph)

The optical characterisation of the ceiling-mounted 405-nm light EDS, operating in ‘blue-only’ mode within a vacant 3 × 3 × 2 m area produced a total of 343 readings, presented in Fig. 2 as 2D slices at each distance measured in the Z direction. The EDS emitted irradiance values across the area ranging from 0.001 – 2.066 mW cm^-2^. The highest irradiance value was collected at the closest measurement taken to the EDS (directly under the light source at a distance of 0.5 m) and the lowest irradiance values were generally collected at maximum distances in X and Y directions from the light source. The range of irradiance values collected was found to decrease as distance from the light source increased in the Z direction; highlighting, as expected, the uniformity of light distribution increases as distance from the light source increases. At a distance of 0.5 m from the light source in the Z direction, the range was found to be 2.06 mW cm^-2^. This value decreased to 0.98 mW cm^-2^ at a distance of 1 m, 0.45 mW cm^-2^ at a distance of 1.5 m and 0.25 mW cm^-2^ at a distance of 2 m. Interestingly, despite variations in light distribution, there was no significant difference (P=0.995) found between the mean irradiance values at each distance in the Z direction (varied between 0.07-0.08 mW cm^-2^).

**Fig. 3:**
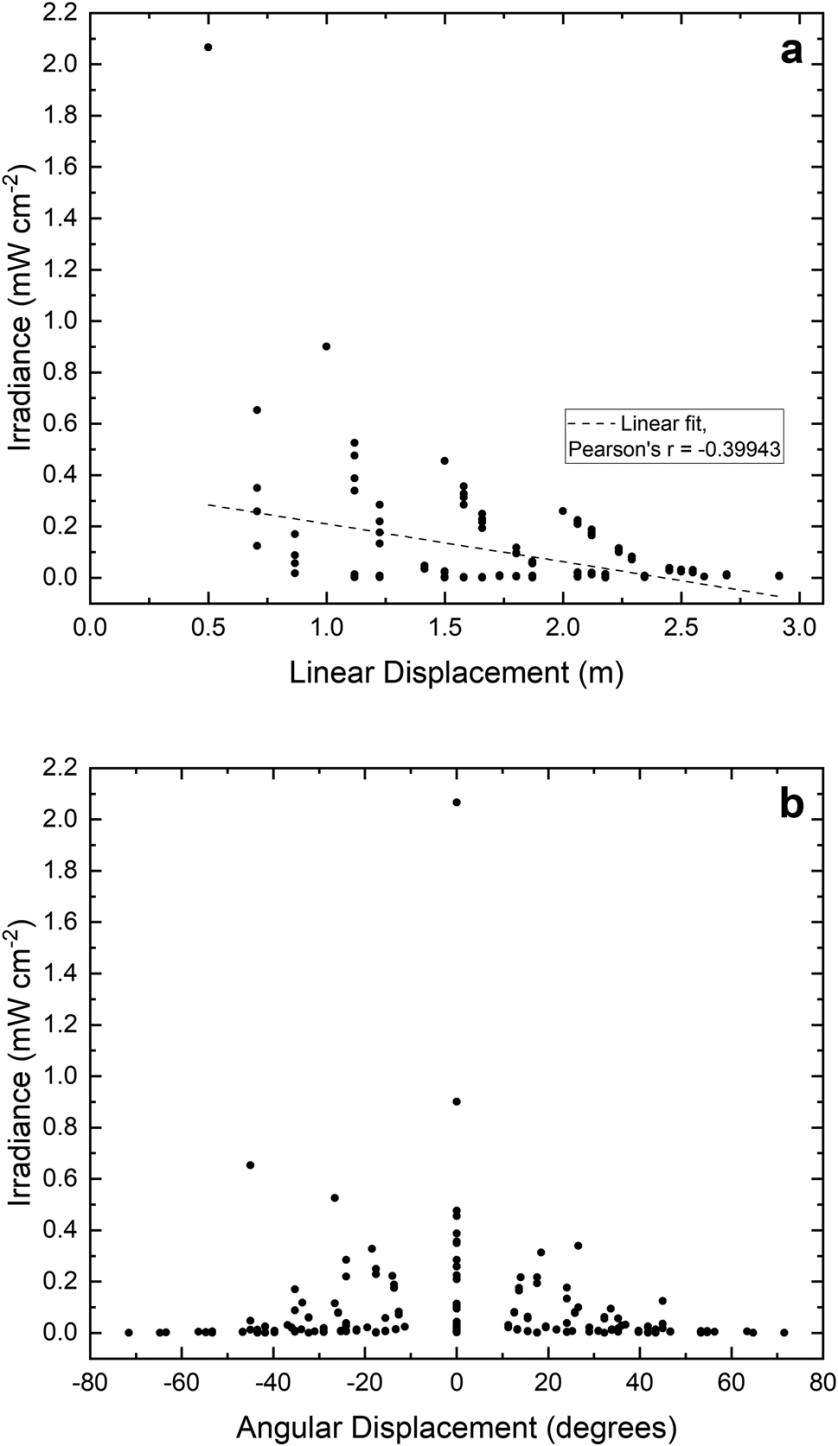
Irradiance distribution within 3 × 3 × 2 m area plotted as a function of **a** linear displacement and **b** angular displacement (in X and Y directions) from the ceiling-mounted 405-nm light EDS

For further analysis of irradiance distribution, the relationship between irradiance and both linear and angular displacement from the light source was determined and is presented in Fig. 3a and b, respectively. Results in Fig. 3a demonstrate a weak, yet significant (P<0.0001), negative linear relationship between irradiance levels and distance from the light source, confirmed by linear regression analysis indicating a Pearson’s r value of -0.39943. General trends in Fig. 3b indicate that the highest irradiance levels produced by the 405-nm light EDS were recorded when angular displacement from the light source in X and Y directions was minimal, with irradiance levels decreasing as angular displacement from the light source increased. Analysis of the data demonstrated a normal distribution of values, with a slight positive skew (value of 6.94).

### Inactivation kinetics of bacteria exposed to 405-nm light EDS

**Fig. 4:**
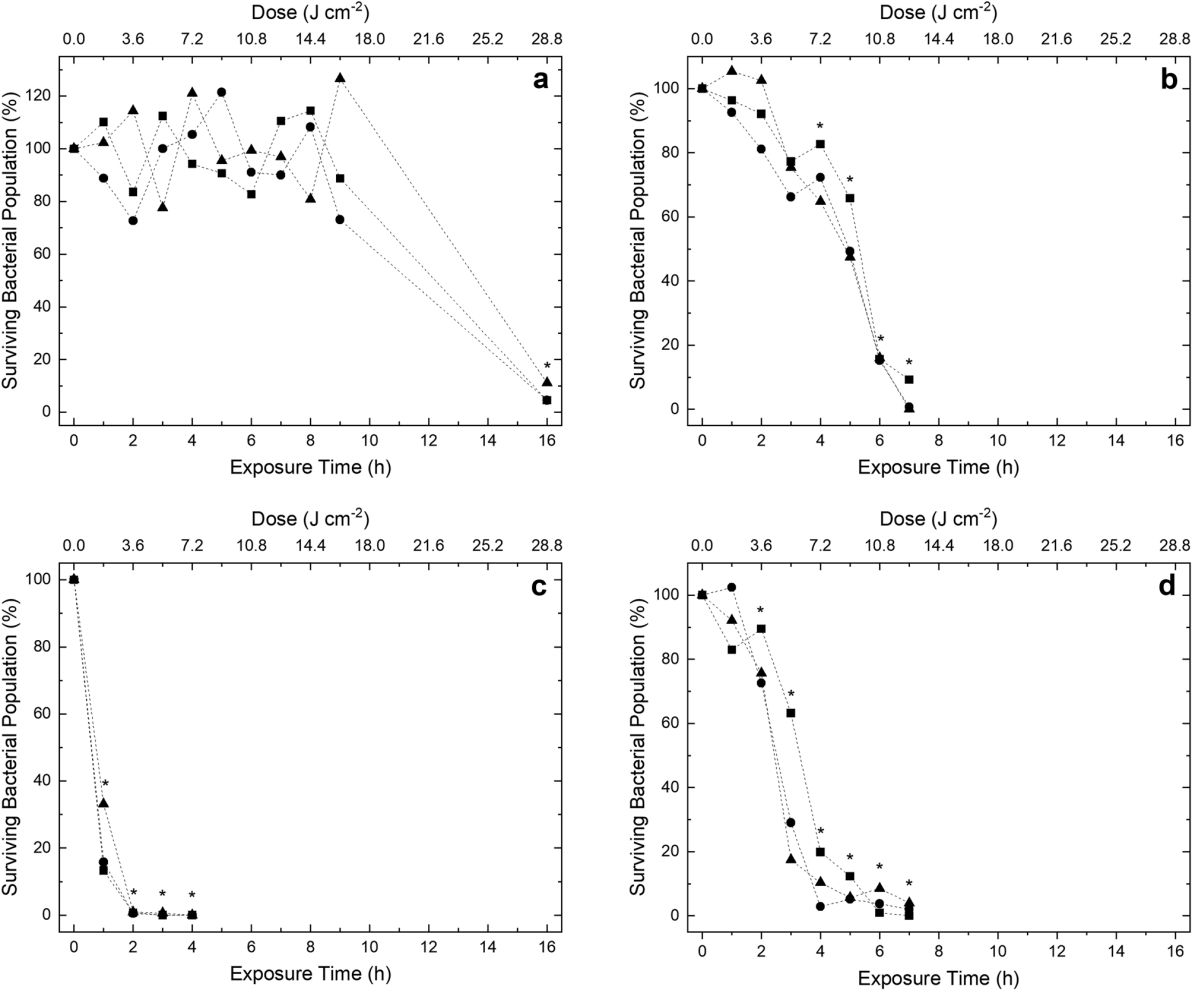
Inactivation of a range of Gram-positive bacterial pathogens associated with hospital-acquired and foodborne infections: **a** *Enterococcus faecium*, **b** *Listeria monocytogenes*, **c** *Staphylococcus aureus* and **d** *Staphylococcus epidermidis*. Bacterial species were seeded onto agar surfaces and exposed to ~0.5 mWcm^-2^ 405 nm light. Experiments were run in triplicate (● run 1; ■ run 2; ▲ run 3). Asterisks (*) represents points where the triplicate CFU plate^-1^ counts were significantly different between test and control samples (P<0.05)

**Fig. 5:**
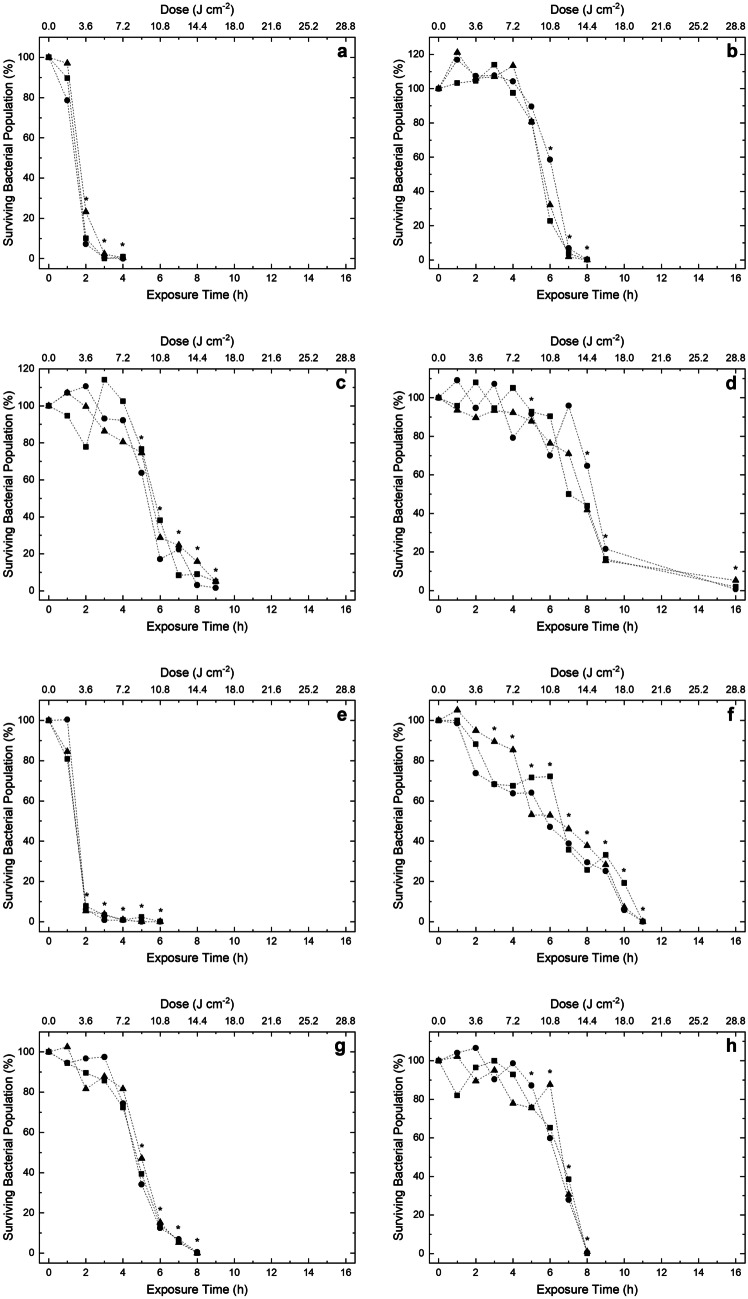
Inactivation of a range of Gram-negative bacterial pathogens associated with hospital-acquired and foodborne infections: **a** *Acinetobacter baumannii*, **b** *Enterobacter cloacae*, **c** *Escherichia coli*, **d** *Klebsiella pneumoniae*, **e** *Pseudomonas aeruginosa*, **f** *Salmonella enterica*, **g** *Shigella sonnei* and **h** *Yersinia entercolitica*. Bacterial species were seeded onto agar surfaces and exposed to ~0.5 mWcm^-2^ 405 nm light. Experiments were run in triplicate (● run 1; ■ run 2; ▲ run 3). Asterisks (*) represents points where the triplicate CFU plate^-1^ counts were significantly different between test and control samples (P<0.05)

Figures 4 and 5 display the inactivation kinetics of four Gram-positive and eight Gram-negative bacterial species, respectively, each seeded onto agar surfaces at an initial population density of 10^2^ CFU plate^-1^ and exposed to low irradiance 405-nm light of approximately 0.5 mW cm^-2^. Results demonstrate complete/near-complete inactivation (≥93.28%) of all species, with general trends indicating that greater levels of inactivation were observed as exposure time increased. Of the Gram-positive species investigated, *S. aureus* (Fig. 4c) was found to be the most susceptible. The organism displayed exponentially decreasing inactivation kinetics with a significant 79.3% mean reduction (P=0.001) observed after 1 h/1.8 J cm^-2^ exposure, and near-complete inactivation (99.3%) achieved after 2 h/3.6 J cm^-2^ exposure. Both *L. monocytogenes* (Fig. 4b) and *S. epidermidis* (Fig. 4d) appeared to follow broadly linear and somewhat sigmoidal shaped kinetics, each requiring 7 h/12.6 J cm^-2^ exposure to achieve near-complete reductions (96.7% and 98.0%, respectively). Results indicate *E. faecium* (Fig. 4a) was the least susceptible, requiring 16 h/28.8 J cm^-2^ to achieve similar levels of reduction (93.3%). Collectively, the Gram-negative species, similar to the Gram-positive species, demonstrated broadly linear, and sometimes sigmoidal, inactivation curves with tailing observed. *A. baumannii* (Fig. 5a) and *P. aeruginosa* (Fig. 5e) were found to be the most susceptible, each requiring 2 h/3.6 J cm^-2^ exposure to achieve significant (P< 0.0001) reductions of 86.5% and 93.4%, respectively. In contrast, *K. pneumoniae* (Fig. 5d) was shown to be the least susceptible Gram-negative species, requiring 8 h/14.4 J cm^-2^ exposure to achieve a significant (P=0.004) 21.1% reduction, and 16 h/28.8 J cm^-2^ to achieve near-complete inactivation (97.4%). In all cases, bacterial contamination in non-exposed control populations displayed no significant decrease throughout the treatment duration.

### Effect of exposure distance and irradiance on inactivation efficacy

**Fig. 6:**
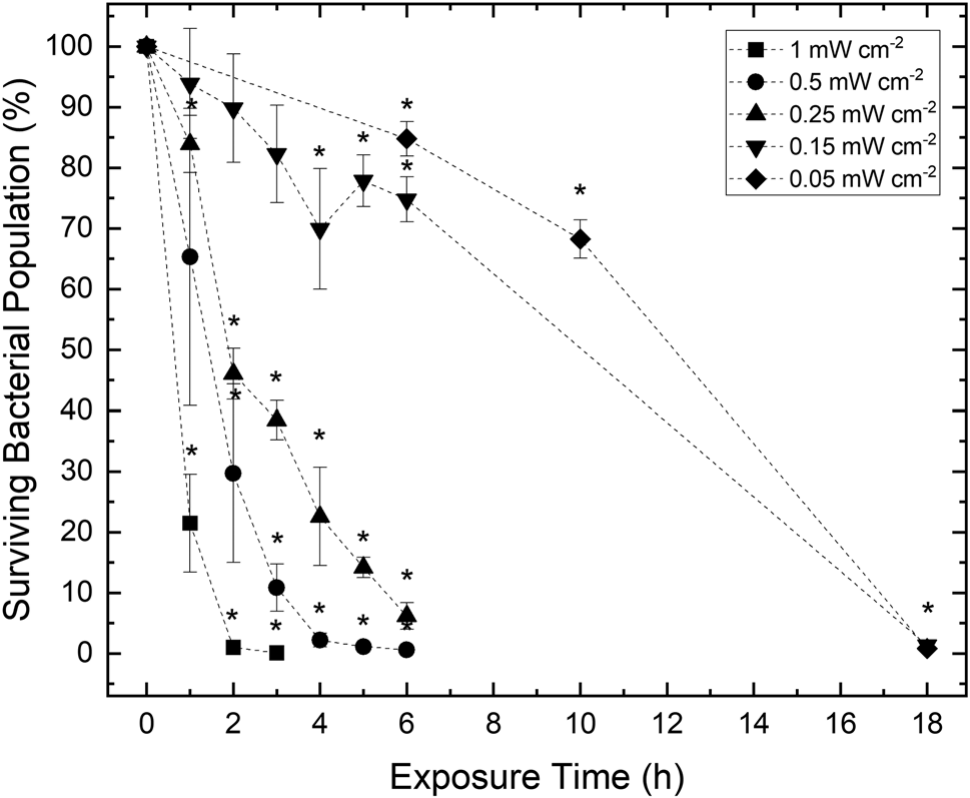
Inactivation kinetics of *Staphylococcus aureus* seeded on agar surfaces and exposed to 405-nm light at irradiances of 0.05, 0.15, 0.25, 0.5 and 1 mW cm^-2^. Each data point represents the mean value ± SD (n = 6). Asterisks (*) represents points where the triplicate CFU plate^-1^ counts were significantly different between test and control samples (P<0.05)

Results in Figure 6 present the inactivation kinetics of *S. aureus* exposed on agar surfaces to the 405-nm light EDS at varying irradiances (0.05-1 mW cm^-2^).

In all cases, a significant downward trend in surviving *S. aureus* populations was demonstrated as the exposure time increased, and non-exposed control samples showed no significant change throughout treatment (P>0.05). Furthermore, general trends indicate that, to achieve significant levels of bacterial reduction, considerably shorter exposure times were required when exposed at higher irradiances; however, less energy was required on a per unit dose basis when exposed at lower irradiances. Exposed at the highest irradiance of 1 mW cm^-2^ for 1 h (3.6 J cm^-2^), a significant 78.53% reduction in comparison to non-exposed control populations was observed (P=0.002). In contrast, when exposed at half this irradiance (0.5 mW cm^-2^), double this exposure time (2 h; 3.6 J cm^-2^) was required to achieve a significant reduction of 70.3% (P=0.008). Likewise, when exposed at the lowest irradiances of 0.15 and 0.05 mW cm^-2^, greater exposure times of 4 h (2.16 J cm^-2^) and 6 h (1.08 J cm^-2^) were required to achieve significant reductions of 30.1% (P=0.025) and 15.2% (P=0.019), respectively. Furthermore, the exposure times required to achieve near-complete inactivation were shown to be significantly shorter when higher irradiance light sources were used (P≤0.05): 2 h (7.2 J cm^-2^) was required to achieve a 99.0% reduction at the highest irradiance of 1 mW cm^-2^; 4 h (7.2 J cm^-2^) and 6 h (5.4 J cm^-2^) were required to achieve similar reductions at irradiances of 0.5 and 0.25 mW cm^-2^, respectively, and up to 18 h was required to achieve similar levels of reduction at the two lowest irradiances (0.15 and 0.05 mW cm^-2^; equivalent to doses of 9.72 J cm^-2^ and 3.24 J cm^-2^, respectively).

**Fig. 7:**
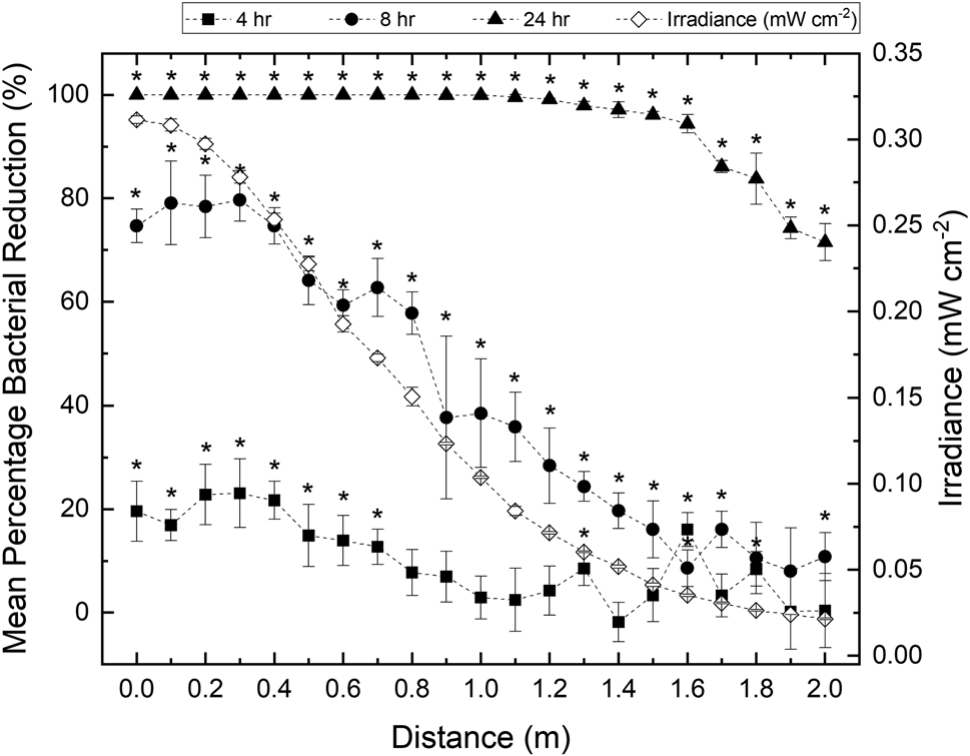
Inactivation kinetics of *Staphylococcus aureus* seeded on agar surfaces and exposed to a low irradiance 405-nm light source at distances ranging from directly below the light source (0 m) up to 2 m. Each data point represents the mean value ± SD (n = 6). Asterisks (*) represents points where the triplicate CFU plate^-1^ counts were significantly different between test and control samples (P<0.05)

Results in Figure 7 show the inactivation kinetics of *S. aureus* on agar surfaces upon exposure to the 405-nm light EDS. The seeded plates were positioned approximately 1.6 m below the ceiling-mounted light source, at varying radial distances starting from directly underneath the centre of the light source (∆s = 1.6 m; $\Delta$ ϴ = 0) up to 2 m (∆s = 2.56 m; $\Delta$ ϴ = 51.3), and were each exposed for durations of 4, 8 or 24 h.

Results comprehensively demonstrate that as the distance from the light source is increased, irradiance decreases, which in turn results in a decrease in the level of inactivation achieved. However, results also demonstrate that significantly greater bacterial reductions occurred as the exposure time to a particular irradiance increased (P<0.0001). Non-exposed control populations showed no significant change throughout treatment (P>0.05).

When *S. aureus* samples were positioned directly below the light source (providing an approximate irradiance of 0.311 mW cm^-2^), reductions of 19.6, 74.7 and 100% were achieved after exposure to the light source for 4, 8 and 24 h, respectively. In contrast, when the samples were positioned at this same distance of 1.6 m below the light source, but at 2 m off centre (providing an approximate irradiance of 0.021 mW cm^-2^), reductions of 0.37, 10.79 and 71.51% were achieved after exposure to the light source for 4, 8 and 24 h, respectively.

Following a 4 h exposure, results demonstrate that significant levels of inactivation (reductions ranging from 12.7-23.1%; P<0.05) were observed at distances ranging from directly below the light source to 0.7 m off centre, where samples were illuminated at irradiances ranging from 0.17-0.31 mW cm^-2^. Exceeding this distance, where irradiances illuminating the samples were ≤0.15 mW cm^-2^, no significant reductions in *S. aureus* populations were observed, with the exception of exposures at distances of 1.3, 1.6 and 1.8 m, where it is assumed that significant differences (P=0.021, 0.003 and 0.023, respectively) were obtained due to sample variability. When the exposure time was increased to 8 h, significant levels of inactivation (reductions ranging from 8.6-79.6%; P<0.05) were observed at all distances up to 1.7 m from the light source, whereby irradiances illuminating samples was as low as 0.03 mW cm^-2^. Exposures at a distance of 1.8 and 1.9 m resulted in reductions of 10.52% (P=0.085) and 8.03% (P=0.223), respectively; however, exposure at a distance of 2 m resulted in a reduction of 10.8% which was found to be significantly different from the non-exposed control populations (P=0.026). Following the longest exposure time of 24 h, significant *S. aureus* reductions (71.5-100%; P<0.0001) were observed at all distances from the light source in comparison to non-exposed equivalent control populations.

## Discussion

This study has successfully characterised the optical output profile of a ceiling-mounted low irradiance 405-nm light EDS within a typical room setting and effectively demonstrated the broad-spectrum efficacy of 405-nm light employed at these low irradiance levels against a range of significant hospital-acquired and food/water-borne infection-causing bacteria, at populations comparable with typical contamination levels found on indoor environmental surfaces.

It is widely acknowledged that bacterial contamination of environmental surfaces plays a significant role in the indirect transmission of infection within settings such as healthcare facilities and the food industry [33, 34]. Physical cleaning within these environments is imperative, however, due to routine working practice, there is continual generation of environmental contamination in the air and on surfaces between cleans which can be further exacerbated by activities such as bed/dressing changes and food processing operations [35, 36]. As such, the ability to implement an infection control technology which addresses bacterial generation and dispersal in real-time is of significant interest. As discussed, recent publications have established the efficacy of 405-nm light EDS units to reduce bacterial contamination levels within clinical settings [23, 26–29]. These studies have successfully highlighted the practical efficacy of these systems within dynamic ward environments, where contamination levels are likely to vary with room activity [36] and the pathogenic potential of environmental bacteria is largely unknown. The inactivation efficacy of low irradiance 405-nm light, like that produced by these systems, for the inactivation of key infection-inducing bacteria, however, is yet to be assessed. Accordingly, this study evaluated the efficacy of low-irradiance 405-nm light, within the range of irradiance levels produced across whole-room environments, for the inactivation of surface deposited bacterial pathogens in a controlled laboratory setting.

The optical output profile of a 405-nm light EDS unit was initially characterised to enhance knowledge of the typical irradiance levels likely to illuminate surfaces and the air in the exposed environment. The dimensions of the profiled area were selected in accordance with Health Building Note 00-03, which advises that single-bed rooms, and each bed space in multi-bed rooms, within general NHS clinical spaces should be between 3-4 m in both length and diameter to enable adequate space for appropriate facilities and necessary room activities [37]. Of the irradiance levels produced within this area (0.001-2.016 mW cm^-2^), 80.6% of measurements were found to be <0.1 mW cm^-2^, 7.1% were 0.1-0.2 mW cm^-2^, 6.1% were 0.2-0.3 mW cm^-2^, 3.1% were 0.3-0.4 mW cm^-2^ and 3.1% were 0.4-2.1 mW cm^-2^ (Figure 2). In all cases, irradiance levels were found to be highest in the areas directly underneath the light source, and lowest at the outermost points measured. For further analysis, the relationship between irradiance and both linear and angular displacement from the light source was determined (Figures 3a and b, respectively). Results in Figure 3a indicate that irradiance output decreased linearly as linear displacement from the EDS increased. As demonstrated in Figure 3b, light irradiance distribution patterns are strongly influenced by angular displacement from the 405-nm light EDS, with general trends indicating a decrease in irradiance output as angular displacement from the unit increases in both X and Y directions. It is important to consider the influence of both linear and angular displacement from the light source, as it may be the case that numerous light sources are required to achieve sufficient levels of decontamination across larger areas. In addition, it should be noted that only surfaces which are directly or reflectively illuminated by the 405-nm light EDS will be treated, and thus it is important to emphasise that this technology should be utilised as a complementary approach in conjunction with established cleaning procedures. Nevertheless, these findings provide an indication of the typical irradiance levels produced spatially by these systems, and thus a greater understanding of the illumination that can be expected to expose pathogens within the environment.

To investigate the broad-spectrum bactericidal efficacy of the 405-nm light EDS, bacteria were exposed at a distance of approximately 1.5 m from the light source, which corresponded to an irradiance of 0.5 mW cm^-2^. This was selected to represent the approximate distance between the room ceiling and frequently touched, thus likely to be contaminated, surfaces within a typical room environment. Bacteria were exposed whilst seeded onto surfaces, rather than in liquid suspension, to mimic surface contamination within occupied environments. The ability of 405-nm light to successfully inactivate high microbial population densities in liquid suspension has previously been indicated in studies by Maclean et al. [8], which showed a 9-log_10_ reduction of *S. aureus*, and Dai et al. [38], which demonstrated an approximate 8-log_10_ reduction of *P. aeruginosa*; however, this study focussed on the inactivation of low-density bacterial populations (10^2^ CFU plate^-1^ / 2-5 CFU cm^-2^) to replicate the typical levels of bacterial contamination likely to be found on environmental surfaces [28]. In addition, this current study employed low irradiance levels and long exposure times to reflect those used by 405-nm light EDS units within occupied settings to achieve a disinfection effect. As previously mentioned, 405-nm light EDS units are designed specifically for continuous use in the presence of people, and thus require low irradiance levels, in the region of those used in the present study, to be safely employed. A more rapid inactivation effect can be achieved with use of higher irradiances, as detailed in previous studies which use irradiances in the region of 10’s – 100’s mW cm^-2^ for bacterial inactivation [2, 8, 10, 19, 20]. However, this study aimed to elucidate the efficacy of low irradiance (<1 mW cm^-2^) 405-nm light under conditions representative of those used for whole-room decontamination.

Results in Figures 4 and 5 demonstrate that exposure to a 405-nm light EDS at an irradiance of ~0.5 mW cm^-2^ successfully inactivated all bacteria contaminated on agar surfaces, with complete/near complete inactivation (~2 log_10_ reductions; ≥93.28%) achieved following delivered doses of 3.6-28.8 J cm^-2^. The organisms selected in this study represent seven of the twelve global WHO priority pathogens, including all of those appointed critical status, which together pose the greatest threat to human health and urgently require novel therapeutics [39]. The findings of this study provide evidence to demonstrate the susceptibility of these key bacterial pathogens to inactivation by the 405-nm light EDS within practical exposure periods. The demonstration that low irradiance 405-nm light has wide antimicrobial activity against all tested organisms within a typical day of use indicates that, in addition to being applicable for environmental disinfection of clinical environments as shown previously [23, 26–29], 405-nm light could also be employed for disinfection within the food industry, and indeed for more widespread disinfection applications associated with environmental cleaning and public health.

Comparatively, the Gram-positive and Gram-negative species investigated demonstrated relatively similar patterns of inactivation: Gram-positive bacteria required 2-7 h exposure for near-complete reductions (≥96.7%), with the exception of *E. faecium*, which required 16 h exposure for a 93.3% reduction; and the Gram-negative bacteria required 3-9 h to achieve near-complete reductions (≥96.1%), with the exception of *K. pneumoniae* and *S. enterica* which required 16 and 11 h for similar reductions (≥97.4%), respectively. No significant difference between the inactivation efficiency of Gram-positive versus Gram-negative species was demonstrated (P=0.901). Interestingly, this contrasts with previous studies investigating 405-nm light susceptibility of high population bacterial densities (>10^5^ CFU ml^-1^) in liquid suspension, which reported an increase in the susceptibility of Gram-positive versus Gram-negative organisms [8, 10, 40]. However, in these previous studies bacteria were exposed at high irradiance levels in non-nutritious diluents for usually short exposure periods (min rather than h) whereas in the present study, low irradiances were used to inactivate low density populations, seeded onto nutritious surfaces and exposed over longer periods (up to 16 h in some cases). It is highly likely that such major differences in exposure conditions will have had metabolic and physiological effects on exposed bacteria and such factors might have affected the bacterial susceptibility to 405-nm light.

Exposure conditions play a significant role in microbial susceptibility to 405-nm light treatment, with recent studies demonstrating an enhancement effect observed when exposed on inert surfaces, including PVC and acrylic, as opposed to nutritious agar surfaces [10], and when exposed in biological media, including saliva, blood plasma and faeces, as opposed to minimal media [15, 16, 41]. The exposure conditions on indoor environmental surfaces are extremely variable, ranging from being completely clean to being contaminated with variable levels of organic and inorganic residues, and so this study utilised general purpose nutrient agar to establish a highly reproducible baseline of the bacterial inactivation which can be achieved using the 405-nm light EDS. Although nutritious media may contain photosensitive material which could augment the 405-nm light inactivation process, it also provides a more nutritious environment and supports the growth of a broad-spectrum of bacteria and so is likely to provide greater protection from oxidative stress than that of inert surfaces. Accordingly, it is expected that bacterial contamination, when present either on clean inert surfaces or in biological residues e.g. bacterial cells in saliva droplets, will demonstrate even greater susceptibility than that shown here, and so future work to assess this will be important to fully evaluate the efficacy of this technology.

General trends indicate that *A. baumannii*, *S. aureus* and *P. aeruginosa* were the most susceptible to inactivation, each requiring 2-3 h exposure for a 1 log_10_ reduction, whilst *E. faecium, K. pneumoniae* and *S.* *enterica* were the least susceptible, each requiring longer exposures of 10–16 h for similar reductions. These findings can be considered consistent with previous 405-nm light inactivation studies. Maclean et al. [8] investigated the susceptibility of a range of medically important bacteria and found *S. aureus* the most susceptible to inactivation, with *K. pneumoniae*, *E. coli* and *E. faecalis* requiring higher doses for a 1 log_10_ reduction. Interestingly, in this previous study, *P. aeruginosa* was found to be one of the more resilient species to inactivation, but this was not the case in this present study, possibly due to differences in the exposure conditions. Interestingly, a more recent study by Hoenes et al. [42] investigated the susceptibility of the ESKAPE pathogens in liquid suspension (10^7^-10^8^ CFU mL^-1^) and found, similar to this present study, that *A. baumannii*, *S. aureus* and *P. aeruginosa* required the lowest doses to achieve a 1 log_10_ reduction. Also, similar to the present study, the authors noted that *E. faecium* and *K. pneumoniae* were the least susceptible to treatment, and that *E. coli* was less susceptible than other strains [42]. Murdoch et al. [10] exposed a range of bacteria also tested in this study (*S. enterica*, *S. sonnei*, *E. coli* and *L. monocytogenes*) on agar surfaces and found *L. monocytogenes* to be the most susceptible, requiring a dose of 180 J cm^-2^ to achieve complete inactivation (2.25 log_10_ reduction), with *S. enterica*, *S. sonnei* and *E. coli* each requiring 1.5 times this dose (270 J cm^-2^) to achieve similar 2.10-2.28 log_10_ reductions. The relative susceptibilities were comparable to that of this study, which found that *S. enterica*, *S. sonnei* and *E. coli* each required a dose 1.6, 1.1 and 1.3 times that of *L. monocytogenes*, respectively, to achieve complete/ near-complete reductions (≥95%).

The aforementioned studies utilised higher irradiances than that employed here (10-71 mW cm^-2^ compared to 0.5 mW cm^-2^), suggesting the comparative susceptibility of each species to light treatment is independent of light irradiance application. In addition to this, these studies required substantially higher doses than those utilised in the present study to achieve similar reductions. In this study, ~2 log_10_ reductions were achieved with doses as little as 3.6 J cm^-2^ (2 h exposure to 0.5 mW cm^-2^). In contrast, Murdoch et al. [10], who employed an irradiance of 71 mW cm^-2^ for low-density surface-seeded populations, required doses in the region of 180-270 J cm^-2^ to achieve similar reductions. Although direct comparisons cannot be made between the results of this study and those of high-density liquid-suspended populations, it is worth noting that Maclean et al. [8] required 27 J cm^-2^ for a 2 log_10_ reduction of *S. aureus*, whilst just 3.6 J cm^-2^ was required here, and Hoenes et al. [42] required 525 J cm^-2^ for a 1 log_10_ reduction of *E. faecium*, whilst just 28.8 J cm^-2^ was required here; with both studies utilising 10 mW cm^-2^ for exposures.

Given that the irradiances produced across a standard room setting were shown to vary between 0.001- 2.066 mW cm^-2^ (Figure 2), five light irradiances (0.05, 0.25, 0.5, 0.75 and 1 mW cm^-2^) within this range were selected for exposure to determine the effects of varying low levels of irradiance on bacterial inactivation. Figure 6 demonstrates successful inactivation of *S. aureus* in all cases, with greater levels achieved as light dose was increased. Results highlight that considerably shorter treatment times were required to achieve significant levels of bacterial reduction when exposed at higher irradiances: an initial significant degree of reduction and near-complete reduction took approximately six and nine times longer, respectively, when exposed at the lowest irradiance (0.05 mW cm^-2^) compared to the highest irradiance (1 mW cm^-2^). Interestingly, results also demonstrate, per unit dose, an increased efficacy of inactivation when exposed at lower irradiances: approximately 3.3 times lower dose was required to achieve significant levels of bacterial reduction and approximately 2.2 times lower dose was required to achieve near-complete reductions when exposed at the lowest irradiance (0.05 mW cm^-2^) compared to the highest irradiance (1 mW cm^-2^).

On this basis, it is possible that lower irradiance 405-nm light, similar to that employed by the 405-nm light EDS, may be more efficient in comparison to that of higher irradiance exposures for the inactivation of bacteria on a per unit dose basis. Sinclair et al*.* [41] recently demonstrated the enhanced efficacy of using a 405-nm light EDS, at irradiances similar to that employed in this study (0.5 mW cm^-2^), for the inactivation of a SARS-CoV-2 surrogate, in comparison to higher irradiance (50 mW cm^-2^) 405-nm light sources; achieving up to 5.8 times greater log_10_ reductions with up to 28-fold greater germicidal efficiency. It is possible that these differences may be due to the specific energy levels required to induce photoexcitation of porphyrin molecules within exposed bacterial cells, and that the use of higher irradiances may be inefficient due to the porphyrin photoexcitation pathway becoming saturated in the presence of excess photons which may not contribute to the inactivation process; whereas with lower irradiances the photons may be utilised more effectively with less photon wastage [43]. This mechanism may also explain the less apparent differences in susceptibility between the Gram-positive and Gram-negative bacterial species tested in this study compared to previously published work which used higher irradiances [8, 10, 40]. Although beyond the scope of this study, the potential increase in inactivation efficacy per unit dose when using lower irradiance treatments, similar to that employed by the 405-nm light EDS, is of significant interest as an area of future study.

In addition to determining the broad-spectrum bactericidal efficacy of the 405-nm light EDS at a fixed position, this study evaluated bacterial inactivation kinetics at varying distances from the light source and at varying irradiances which could typically be expected at different positions within a whole-room environment. *S. aureus* was chosen as a model organism for this study as it is one of the most frequent surface contaminants in hospitals [44] and staphylococcal food poisoning is the most prevalent foodborne intoxication to occur globally [45]. Results in Figure 7 demonstrate that the irradiance decreased from approximately 0.311 mW cm^-2^ directly 1.6 m below the light source to 0.021 mW cm^-2^ when placed 2 m off centre (∆s = 2.56 m). The extent of inactivation achieved per unit exposure time was also shown to decrease as distance from the centre of the light source increased, with an initial exposure of 4 h demonstrating a 19.58% reduction directly underneath the light source (0.311 mW cm^-2^ irradiance) in comparison to just a 0.38% reduction underneath the light source at a distance of 2 m off centre (0.021 mW cm^-2^ irradiance). These results agree with previous work by this laboratory demonstrating that inactivation levels significantly decrease as distance from the 405-nm light source increases [32]. Results here do, however, also demonstrate that, regardless of distance from the light source, the level of inactivation achieved increases as the time of exposure is increased. Following an 8 h exposure, significant bacterial reductions were achieved at distances approximately 2.4 times greater than that achieved after 4 h exposure (0.7 m off centre in comparison to 1.7 m off centre; P<0.05). When this was further increased to a 24 h exposure, significant bacterial reductions were demonstrated up to 2 m from the light source: 2.9 times greater than after the original 4 h exposure (P<0.05). A strong correlation between bacterial kill and exposure time to 405-nm light EDS units has previously been demonstrated on various surfaces within clinical settings [23, 27] and this study confirms that pathogenic bacteria exposed under controlled laboratory conditions behave in a similar manner.

Although collectively Figures 6 and 7 indicate that the treatment times required to achieve significant bacterial inactivation are prolonged at lower irradiance, results importantly also demonstrate that inactivation was still achievable at the lowest irradiances investigated and, given the continuous operational nature of the 405-nm light EDS, it is expected that sufficient inactivation would occur even at these lower irradiance exposures; further justifying the utilisation of the 405-nm light EDS for the decontamination of whole-room environments. Given its high safety profile, they can be retrofitted in place of an overhead light source and utilised continuously without posing a disturbance to room activity, with increased inactivation occurring as exposure time is increased.

It is important to mention that the inactivation kinetics presented in this study are representative of organisms which have been cultured under optimal conditions. Bacterial populations were cultured at 37°C in growth media and then exposed to the 405-nm light EDS on nutrient agar surfaces; cultivation and exposure conditions which exert minimal stress on the organisms. Importantly, laboratory studies have demonstrated that stressed organisms show increased susceptibility to 405-nm light inactivation [14]. Thus, when used practically for inactivation of bacterial contamination on environmental surfaces, such as in the case of hospital room disinfection [23, 26–29], inactivation kinetics are likely to be enhanced. Organisms can remain on environmental surfaces for prolonged periods of time, with up to 90 days recorded previously in the case of staphylococci and enterococci [46], but these organisms will be stressed due to desiccation, and in some cases, sublethal exposure to disinfectants, and these concurrent stresses will likely increase microbial susceptibility to decontamination using 405-nm light. By exposing bacteria under controlled laboratory conditions with minimal stress factors, this study importantly demonstrates the likely exposure times required to inactivate key bacterial pathogens under ‘worst-case scenario’ conditions, with inactivation expected to be enhanced when used in practice.

Further considering the work of this study towards practical application in ‘real’ environments, it has been demonstrated here that bacterial contamination on surfaces – in this case, agar surfaces – can be effectively inactivated. However, previous publications have demonstrated that 405-nm light exposure is also effective for inactivation of bacteria on inert surfaces such as glass, acrylic and polyvinyl chloride (PVC) [10]; and indeed the studies demonstrating the efficacy of 405-nm light for disinfection of hospital isolation rooms collected samples from a wide range of surfaces and materials including door handles, table/locker surfaces, bed rails, computer keyboards/mouse and light switches [23, 26–29].

Overall, this study has successfully characterised the optical output profile of a 405-nm light EDS within a typical room setting and demonstrated the broad-spectrum antimicrobial efficacy of 405-nm light employed at low irradiances within this range for the inactivation of low-density populations of surface-seeded bacterial pathogens, known to be associated with healthcare and foodborne-related infections, within a controlled laboratory setting. In addition, successful bacterial inactivation was achieved within exposure times realistic of those employed for whole-room decontamination, and the similarities in inactivation kinetics between Gram-positive and Gram-negative bacteria when using low irradiance light was established. The effects of altering the distance of a bacterial sample from the light source, and thus the irradiance at which it would be illuminated, were considered and, although differing levels of inactivation were demonstrated, complete (~2 log_10_) reductions were still achievable, suggesting these factors will unlikely have a significant impact on the overall inactivation effect achieved. The irradiance levels (≤1 mW cm^-2^) and large exposure distances (up to 2.56 m) used in this study are quite different to anything that has previously been described in the literature, and they have been used in order to provide laboratory-based demonstration of the efficacy of 405-nm light for large scale environmental disinfection applications, as have been detailed by the previous studies carried out in the hospital environment [23, 26–29]. The findings of this study comprehensively advance knowledge of the 405-nm light EDS and its applicability for whole-room decontamination which, combined with its inherent safety benefits, furthers its widespread utilisation across the infection control sector.

## Data Availability

Data supporting this publication are stored by the University of Strathclyde. Details of the data and how it can be accessed are available from the University of Strathclyde Knowledge Base at https://doi.org/10.15129/85368aa1-ab78-4abd-8874-7e39e0769838.
